# Are companies responding to minimum wage increases by reducing corporate social responsibility?

**DOI:** 10.1371/journal.pone.0313225

**Published:** 2025-01-06

**Authors:** Hao Wang, Tao Zhang, Xi Wang

**Affiliations:** 1 Faculty of Humanities and Social Sciences, Macao Polytechnic University, Macao, China; 2 Rua de Luís Gonzaga Gomes, Macau, China; Southwestern University of Finance and Economics, CHINA

## Abstract

Corporate social responsibility (CSR) has been widely discussed. However, the existing literature does not delve into the theoretical mechanism to show how companies adjust their CSR in the face of minimum wage increases. This may be due to the lack of a theoretical framework that clarifies the relationship between minimum wage increases and CSR adjustments. The objectives of this study is to fill this gap by investigating the impact of minimum wage increases on CSR, employing both cost stickiness and optimal distinctiveness theories. We use the data from the CSMAR database, the Human Resources and Social Security Administration, and Hexun rating system. The subject of this study is China’s A-share listed companies during 2010–2020. This study employs fixed-effects models for a panel data. The findings reveal that minimum wage increases are significantly associated with a reduction in both strategic CSR and responsive CSR. Notably, the decrease in responsive CSR outweighs that of strategic CSR. Furthermore, our results indicate that customer concentration or CSR sensitivity significantly moderates this relationship. More particularly, firms with higher customer concentration are less responsive to minimum wage increases in their CSR activities. Firms with higher sensitivity in CSR are more likely to mitigate the reducing effect of the minimum wage on CSR. By revealing how minimum wage increases affect CSR and its economic consequences, our study provides scientific recommendations for policymakers to measure the impact of minimum wage policies at the firm level.

## 1 Introduction

Corporate Social Responsibility (CSR) represents a pivotal strategic endeavor for businesses, embodying the practice of aligning with prevailing norms, expectations, and practices to cultivate legitimacy and secure resource support [1–3]. Simultaneously, it facilitates the preservation of a distinct competitive edge through the prioritization of scarce resources, enhancement of cost management, and formulation of strategic investment approaches [4–6]. Academics have extensively debated a critical paradox: the challenge of how organizations can meet the basic legal requirements to access government resources while simultaneously cultivating their strategic assets to distinguish themselves and achieve a competitive advantage [4–6]. Much of the existing literature on achieving optimal distinctiveness at the firm level has concentrated on strategies for maximizing a firm’s legitimate uniqueness [7–10]. In parallel, CSR research has predominantly focused on how organizations can fine-tune their CSR investment strategies to navigate institutional pressures and sustain strategic uniqueness [1, 8].

As a fundamental policy for worker protection, the establishment of minimum wage standards has emerged as a common institutional practice worldwide. It was not until the Minimum Wage Regulations were introduced in 2004 that China began to vigorously promote and enforce a minimum wage system [11]. From 2004 to 2020, China’s average monthly minimum wage witnessed a significant surge, escalating from 430.75 yuan to 1775.16 yuan, a 412.11-fold increase [11]. This remarkable growth in the minimum wage serves as a pivotal means for evaluating a firm’s capability in cost management and risk mitigation. It provides a crucial framework for a systematic exploration of how to strike a dynamic balance between legitimacy and competitiveness in the process of CSR fulfillment [12, 13].

Investment activities of firms, such as CSR, entail costs [14]. On top of that, any adjustment of investment activities requires the payment of a corresponding adjustment cost, which is cost stickiness. The greater the cost stickiness, the less likely the investment activity will be adjusted [15]. The impact of minimum wage increases on CSR investment is complex and remains uncertain from the perspective of cost stickiness. The scholarly discussion on CSR investment identifies one critical shortcoming: a limited examination of CSR investments from a cost perspective [14, 15]. It is essential to delve into the internal mechanisms driving changes in CSR, and this paper aims to shed light on this issue by applying the theory of cost stickiness.

Porter and Kramer’s CSR decision-making framework, which distinguishes between responsive and strategic CSR based on firms’ motivations for legitimacy and differentiation, serves as a foundation for our analysis [16, 17]. Responsive CSR, which focuses on improving short-term relationships, is considered a form of symbolic management and short-term endeavor that is not central to the business’s core operations [15, 18]. We propose that because of its lower cost stickiness, responsive CSR is less critical to a firm’s fundamental interests and its marginal benefits are unlikely to enhance the firm’s core competitiveness. Moreover, the additional effects of responsive CSR do not compensate for long-term labor cost increases, positioning it as a potential target for cost reductions by managers.

On the other hand, strategic CSR represents a long-term commitment that requires significant resources. We argue that managers may need to implement substantial changes in management structures to improve organization and compensate for the loss of vital resources [15, 18]. Owing to the high cost stickiness associated with strategic CSR, firms facing labor cost pressures should meticulously evaluate the consequences of reducing strategic CSR investments [15, 18]. Such considerations include potential economic losses, social costs, contractual or psychological costs, and the diminishment of intangible assets like reputation capital, before making decisions on cutbacks [19–22].

Previous studies have documented attempts by managers to mitigate cost pressures by scaling back on CSR investments [22–24]. This approach, however, raises concerns due to the neglect of adjustment costs associated with changes in CSR. The challenges of rebuilding trust relationships and the erosion of industry identity may result in indirect losses and potential crises for businesses [25, 26]. Additionally, in response to the expectations of consumers, employees, channel partners, and regulatory bodies, an increasing number of managers recognize the additional benefits and intangible value provided by CSR as essential compensations for rising labor costs. This recognition leads to the belief that managers will not only sustain their CSR initiatives but may also intensify efforts in areas such as strategic partnership development, enhancement of firm reputation, and management of crucial customer information. This perspective underscores the importance of viewing CSR investments not merely as a cost but as a strategic asset that can offer significant returns, particularly in managing and offsetting labor cost increases [1, 20, 24, 27–35].

Overall, there are some gaps between the minimum wage and CSR cross-sectional studies. Excessive minimum wages may increase the operating costs of firms. But little literature has examined the impact of such cost increases on CSR investments. Similarly, as a business practice, CSR is influenced by numerous factors, but few studies have analyzed the role of minimum wage on CSR. In addition, the policy environment and market conditions in different countries and regions also affect the relationship between minimum wage and CSR practices. Therefore, the purpose of this study is to attempt to explore the relationship between minimum wage and CSR through empirical analysis using Chinese A-share listed companies as examples. And, we expect that the results of our study provide some reference and basis for the government and enterprises to formulate policies.

To bridge above research gaps, the study employs both the theory of cost stickiness and the theory of optimal distinctiveness to examine how minimum wage increases affect managers’ perceptions of responsive and strategic CSR, alongside investigating the mediating roles of customer concentration and CSR sensitivity in this relationship. The study utilized a sample from Chinese listed companies spanning from 2010 to 2020 as our empirical dataset, capitalizing on China’s distinctive institutional landscape as a fertile ground for analyzing the dynamics between institutional pressures and strategic management perspectives. Specifically, the study seeks to unravel the following queries: First, how does the increase in minimum wage influence the allocation towards strategic and responsive CSR? Second, with customer concentration—defined as the share of sales to a company’s top five customers in its total revenue—serving as a measure of a firm’s customer base diversity [36, 37], how does this concept shape the limits of CSR investment variability in light of customer responsibility? Lastly, considering industries’ CSR sensitivity, which denotes the degree to which various sectors give precedence to CSR [38, 39], the purpose of this study is to understand how this heterogeneity affects the modifications in strategic and responsive CSR investments.

Our study endeavors to contribute to the discourse on minimum wages and CSR in several key ways. First, it broadens and deepens the investigation of CSR through the prism of the minimum wage system, offering new insights into how wage policies intersect with corporate social responsibilities. Second, the study aims to refine the ongoing discussion on the motivations driving Chinese firms to meet their CSR obligations by integrating institutional and strategic perspectives, thus shedding light on the complex interplay between external pressures and strategic corporate decisions. Third, the study addresses a significant gap by underlining China’s increasing role in the global economy and the diverse importance that its management places on different CSR dimensions, thereby enhancing our understanding of CSR priorities in varying contexts. Finally, by exploring how customer concentration and industry sensitivity to CSR influence CSR investments, our study reveals the nuanced mechanisms and processes through which minimum wage increases impact CSR initiatives. Through this comprehensive analysis, the study aims to lay the foundation for the development of more effective decision-making strategies that will improve the cost management capabilities of companies and promote innovative approaches to CSR at all levels.

## 2 Conceptual review and hypothesis development

### 2.1 Optimal distinctiveness and CSR

The quest for optimal distinctiveness stands as a pivotal goal in organizational management. Initial research focused on pinpointing a singular, static equilibrium where a firm’s legitimacy and uniqueness simultaneously peaked. However, evolving theories, notably from Zhang et al. (2020) and Zhao et al. (2017), challenge this notion by highlighting the fundamentally pluralistic and dynamic essence of organizations [24, 32]. These insights prompt a shift from static, oversimplified decision-making models towards a more nuanced, multidimensional strategy formulation. This approach, however, often sidesteps the diversity of an organization’s external milieu, concentrating instead on the subtleties of internal strategic maneuvers [32, 40, 41].

In this context, CSR is conceptualized not merely as a series of rigid, uniform protocols or governance edicts, but as a broad management ideology characterized by its engagement with a variety of stakeholders, thereby increasing its complexity [20, 23, 24]. Given this perspective, we argue that the intricate nature of organizational strategy should reflect the multifaceted reality of the external environment. This alignment enables organizations to navigate the delicate balance between maintaining consistency and fostering differentiation, under certain conditions, thus achieving optimal distinctiveness in a dynamic setting.

Some researchers advocate that CSR initiatives are primarily adopted to meet government expectations and conform to societal norms and values [42, 43]. This perspective underscores the strategic formation of institutionalized CSR structures, encompassing frameworks, processes, and dedicated personnel, aimed at affirming a company’s normative, credible, and legitimate presence to the public. The global acknowledgment of CSR’s significance is on the rise, spurring the creation of various standards and national strategies dedicated to fostering, propagating, or setting a more universally accepted global CSR benchmark [44, 45]. Consequently, compliance with governmental standards is increasingly recognized as a pivotal factor for both burgeoning markets and established economies [46, 47].

Conversely, a different group of scholars argues that the essence of corporate endeavors lies in profit maximization, with CSR activities functioning as pivotal instruments for securing a unique competitive advantage. From this vantage point, the corporate aim transcends simple compliance with institutional mandates towards a more nuanced strategy of addressing the distinct demands of customers and other stakeholders. Through innovative products, management approaches, and operational strategies, companies strive to set themselves apart and reap maximal rewards [32]. CSR, in this context, is seen as a lever for accessing scarce resources, advanced technologies, and comprehensive market intelligence, as well as strengthening ties with key stakeholders [48, 49]. We contend that escalating CSR investments not only unveils unique opportunities but also fortifies a company’s competitive position, cementing its marketplace dominance.

Expanding upon previous research and drawing on the insights provided by Porter and Kramer, we propose a dual-dimensional framework for understanding CSR implementation: responsive CSR and strategic CSR [16, 17]. Responsive CSR involves conforming to governmental directives, laws, and regulations, reflecting a firm’s commitment to meeting external obligations and expectations. This dimension of CSR emphasizes the importance of compliance as a foundational element of corporate responsibility [50, 51].

In contrast, strategic CSR goes beyond mere compliance, focusing on the acquisition of unique resources through targeted CSR activities [52, 53]. This approach to CSR is proactive and resource-centric, aiming to leverage CSR initiatives for strategic advantages, such as enhanced reputation, improved stakeholder relations, and access to new markets or resources. By distinguishing between responsive and strategic CSR, we highlight the multifaceted nature of CSR practices and underscore the potential for firms to not only fulfill external demands but also strategically position themselves for long-term success and sustainability.

### 2.2 Cost stickiness of CSR

CSR is widely recognized as a firm’s voluntary commitment to improve social and environmental conditions, aimed at fulfilling stakeholder expectations [54, 55]. This concept has been extensively studied, leading to a variety of interpretations [56]. We subscribe to a widely accepted definition among management scholars, which views CSR as a set of independent actions by firms intended to enhance social well-being [57]. CSR responsibilities span a broad spectrum of initiatives, including promoting environmental sustainability, fostering workforce diversity, improving working conditions, supporting education and housing initiatives, and ensuring the provision of high-quality products.

CSR initiatives are generally perceived as strategic approaches to win stakeholder support, optimize resource allocation, gain positive media coverage, establish community legitimacy, and reduce scrutiny from stakeholders and researchers [58]. By facilitating access to scarce resources, enhancing employee attraction and retention, improving marketing and service, and securing social legitimacy, CSR can have a positive impact on a firm’s performance [59, 60]. Despite the potential benefits, the costs associated with CSR investments prompt varied viewpoints on their prudence [61, 62]. Advocates argue that failing to engage sufficiently in CSR can undermine a firm’s legitimacy, while critics contend that CSR investments are often superficial attempts to enhance corporate image at the expense of shareholder value [58, 63].

Leveraging the theory of cost stickiness, we argue that a significant portion of CSR initiatives are the result of strategic managerial decisions, which makes scaling back on these investments difficult without incurring substantial additional costs. These costs can be categorized as the economic, social, contractual, or psychological sacrifices associated with the process of adjusting investments. This perspective challenges the conventional cost accounting model, which posits that costs vary directly with changes in activity levels, by suggesting that the reality of reducing CSR investments is fraught with high adjustment costs. For example, a hybrid vehicle, which offers environmental benefits over traditional cars, can command a higher market price due to its contribution to reducing pollution [64, 65]. However, in the face of declining sales during an economic downturn, a company might hesitate to cut back or divert resources from hybrid electric vehicles, viewing the slump as temporary. This scenario illustrates the concept of cost stickiness, where the behavior of costs is asymmetric; the costs increase more with an activity level increase than they decrease with a reduction in activity level.

By integrating the theory of optimal distinctiveness with cost stickiness, we conclude that strategic CSR represents a costly and long-term commitment, often involving investments that are difficult to reverse [9]. The high cost stickiness associated with strategic CSR is due to the continuous investment required to meet responsibilities towards employees, customers, and other stakeholders. In contrast, responsive CSR, which aligns more closely with compliance and short-term goals, demands fewer resources and is typically viewed as a short-term investment. This leads to the conclusion that responsive CSR is characterized by lower cost stickiness, reflecting its more flexible and less integral role in a firm’s strategic framework.

### 2.3 CSR adjustment under minimum wage increase shock

Since its inception in 1993 with the Regulations on Minimum Wages for Firms, China has progressively developed its minimum wage system, notably advancing with the 2004 Regulations on Minimum Wages. The pace of minimum wage adjustments has quickened since 2010, with 2015 witnessing a significant expansion in the scope of adjustments amidst economic downturns. Furthermore, 2017 saw substantial increases in provinces already maintaining higher minimum wage levels compared to the previous year [66].

The academic discourse surrounding minimum wage has uncovered several key themes, emphasizing its critical role in safeguarding basic living standards and improving income distribution. Additionally, the literature reveals how minimum wage increases influence the behaviors of minimum wage earners at the micro level [66]. On a macro scale, research tends to focus on the effects of minimum wage on employment and inflation [67, 68], while micro-level studies examine its impacts on human capital [69], corporate innovation [23], environmental stewardship [21, 29, 30], and foreign investments [70–72].

In fact, the relationship between minimum wage and CSR is a complex and multi-layered topic, with the two influencing and complementing each other. The minimum wage system aims to protect the basic living standards of low-income workers, prevent over-exploitation and enhance social equity [66]. CSR, on the other hand, emphasizes that while pursuing economic benefits, enterprises also need to pay attention to social and environmental responsibilities to achieve sustainable development [54, 55]. In addition to the above favorable effects on enterprise development, minimum wage may also bring negative impacts to enterprises. And these related studies are relatively few.

For example, for enterprises, wage costs account for a relatively large proportion of total operating costs, and an excessively high minimum wage may lead to a heavier operational burden on enterprises, thereby affecting their economic efficiency [14, 15]. Some enterprises may respond to rising wage costs by reducing the number of employees, curtailing working hours or lowering other benefits, which to some extent undermines investment in CSR [58, 63].

In addition, if an enterprise is economically stronger, an increase in minimum wage may have less impact on the enterprise, which is more capable of and has more resources to fulfill CSR [59, 60]; whereas for less powerful enterprises, an increase in minimum wage may pose a greater challenge to the survival and development of the enterprise, and the enterprise will not have time to invest in CSR [23, 73, 74].

To summarize, the relationship between minimum wage and CSR is complementary. A reasonable minimum wage system can not only protect the rights and interests of employees and improve the quality of life, but also promote enterprises to better fulfill their CSR and achieve sustainable development. However, faced with the rise of minimum wage, there are still some gaps in the research on how CSR changes need to be explored in China.

In addressing the research gap related to the adjustment mechanism of CSR in the context of rising minimum wages, our exploration centers on the economic implications of strategic and responsive CSR adjustments. Strategic CSR, deeply integrated into a firm’s strategic, resource, capability, and stakeholder frameworks [75–77], particularly focusing on product and employee responsibilities, requires long-term vision, extensive resource commitment, and significant organizational revamps [78–80]. Scholars have identified the potential of strategic CSR to improve management practices, operational efficiency, and product quality, thus accruing strategic assets like reputational or moral capital. Given its intricacies, strategic CSR is characterized by high cost stickiness [76, 77].

Building on Porter and Kramer’s delineation of strategic CSR as the amalgamation of product and employee responsibilities, we posit that the pressure of increased minimum wages is unlikely to prompt managers to drastically cut back on strategic CSR investments [16, 17]. This stance recognizes the entrenched position of strategic CSR within the broader strategic agenda, underlining its significance in maintaining competitive and ethical corporate stature amidst financial pressures.

Responsive CSR is tailored to swiftly satisfy stakeholder expectations, align with current norms and practices, enhance legitimacy, and garner support from governmental resources [81, 82]. It is often perceived as a symbolic action and a transient commitment, disconnected from the core activities of an organization. In the unique institutional landscape of China, commitments to community (such as charitable giving) and environmental stewardship (e.g., investments in environmental protection) stand out as critical approaches to cultivating institutional and environmental legitimacy [80, 83]. These efforts are frequently deployed to gain favor with governmental bodies or as temporary solutions to crises of accountability. Additionally, viewed through the lens of agency theory’s over-investment hypothesis, charitable contributions may be interpreted as manifestations of managerial self-interest, with CEOs potentially favoring philanthropy at the expense of shareholders, thereby negatively impacting firm value and potentially hindering firm growth [79, 84].

Due to its ephemeral nature, ease of reversal, minimal resource demands, and low adjustment costs, responsive CSR is less impacted by the phenomenon of cost stickiness [85, 86]. Thus, it is anticipated that managers will quickly scale down responsive CSR activities in the face of the financial burdens imposed by minimum wage increases. Following Porter and Kramer’s conceptual framework, we identify environmental stewardship and philanthropy as elements of responsive CSR, underscoring their role in addressing immediate stakeholder demands and regulatory expectations without fundamentally embedding these practices into the organization’s strategic core [16, 17].

Based on the above analysis, we propose the following assumptions:

*Hypothesis 1*: When the minimum wage is increased, due to higher cost stickiness, companies will reduce strategic CSR to a lesser extent, while due to lower cost stickiness, they will reduce responsive CSR to a greater extent.

### 2.4 The moderating effect of customer concentration

The prevailing research on determinants of CSR efficacy predominantly examines institutional dynamics, company-specific characteristics, and managerial qualities. Yet, the influence of firm customers—a pivotal stakeholder group—on shaping supplier CSR practices remains underexplored. CSR is commonly perceived as a safeguard against negative incidents. Evidence suggests that firm customers often require their suppliers to adopt CSR practices as a means to avert reputational damage and financial losses that may arise from unethical behaviors on the part of the suppliers [87–90].

Ross (1977) constructed a dividend signal model and was the first to propose the signaling theory [91]. Signaling theory holds that managers can use business activities to convey information about the development of the enterprise to stakeholders in the market [91]. Although information asymmetry in the market is inevitable, and enterprises may face principal-agent problems at any time due to poor information transmission, enterprises can convey positive information about their stable and sound status to stakeholders through investment in CSR, so as to enhance stakeholders’ resource support and investment confidence in the enterprise and improve the enterprise’s ability to resist some shocks in the external environment, such as minimum wage increases [88, 91]. Similarly, the disclosure of CSR can serve as a signal, conveying comprehensive information about production and management to stakeholders, enhancing corporate reputation and strengthening the connection between the company and its stakeholders. Under the impact of the cost increase caused by the minimum wage, the high-quality signal conveyed by CSR can help companies enhance their brand image, gain goodwill, and gain the support of employees [88, 91].

According to signaling theory, CSR initiatives can reduce information asymmetry between customers and suppliers, with customers viewing these activities as signs of the suppliers’ prospective performance [92]. In situations where customer concentration is high, thus granting customers greater bargaining leverage, suppliers tend to be more committed to meeting their customers’ CSR expectations [92–94]. If there is a high concentration of customers served by a business, then the customer’s bargaining power in contract negotiations will increase [88]. That is, from the perspective of the service provider, if the concentration of customers in a company is high, the enterprise providing the service will pay more attention to the needs of customers in order to protect the interests of major customers [88, 89]. Due to the necessity for companies to cater to the needs of major customers, companies will release signals to customers to maintain relationships by fulfilling CSR activities, especially those related to maintaining customer relationships [73, 95]. CSR activities that fulfill customer preferences increase customer loyalty and gain more control over contractual pricing at the cost of approaching flattery [73]. Additionally, a higher customer concentration may increase operational risks for suppliers. Some studies regard CSR practices as a differentiation strategy for enterprises to reduce operational risks, because fulfilling more types of CSR conveys a more comprehensive picture of the enterprise’s operations to the outside world, in the hope of winning the favor of customers other than major customers [73, 90, 95].

Therefore, in the face of the impact of minimum wage increases, enterprises with higher customer concentration may increase their investment in corporate social responsibility as a strategic measure to maintain customer relationships, reduce risks and ensure competitive advantages, compared to enterprises with lower customer concentration.

Based on the above analysis, we can put forward the following hypotheses:

*Hypothesis 2*: Customer concentration mitigates the impact of minimum wage increase on strategic and responsive CSR.

### 2.5 The moderating effect of CSR-sensitivity

Previous research has revealed significant variation across industries, particularly when scrutinizing investment trends in research and development (R&D) and CSR. While firms, on average, maintained their R&D and CSR investments, a detailed analysis identifies exceptions [74]. Specifically, firms within industries characterized by high R&D intensity and low CSR sensitivity have scaled back their investments in these domains [23, 74, 96]. This trend seems reasonable, given that the competitive advantage of firms in these business-to-business (B2B) sectors may not rely as heavily on innovation capabilities or stakeholder engagement [23, 74, 96].

Furthermore, in industries where CSR concerns are less pronounced, companies might be more inclined to reduce their CSR endeavors [97, 98]. Moreover, in areas with lower CSR sensitivity, indicating that stakeholder support is less critical to a firm’s competitive edge and longevity, the strategic importance of CSR is likely to wane [99, 100]. According to the 2001 version of the industry classification by the China Securities Regulatory Commission (CSRC), sectors such as finance, public utilities, and commerce are deemed to have higher CSR sensitivity, while B2B fields, including manufacturing, are considered to have lower CSR sensitivity [74, 96]. Our observations suggest that although firms did not universally decrease their CSR investments during periods of economic downturn, reductions were more prevalent in sectors where innovation and stakeholder relationships are not seen as crucial for maintaining competitive advantage.

Resource-based theory holds that enterprises can restructure and strategically transform by integrating the resources they control, which can help improve competitiveness [101]. Companies that are more sensitive to CSR believe that fulfilling CSR is an important measure to maintain a competitive advantage [96, 101]. By strengthening investment in CSR, enterprises can effectively strengthen their ties with strategic stakeholders such as employees, consumers and suppliers, and in turn obtain scarce resources, such as resources for organizing employee learning and training, resources for customer information, and resources for building partnerships with government departments, community departments and businesses [101, 102].

Similarly, we believe that maintaining investment is more common among enterprises that consider CSR implementation to be crucial for maintaining a competitive advantage, i.e., those that are more sensitive to CSR, even in the face of the impact of rising operating costs due to minimum wage increases.

Based on the above analysis, we can put forward the following hypotheses:

*Hypothesis 3*: *CSR-sensitivity industries mitigate the impact of minimum wage increase on strategic and responsive CSR*.

## 3 Data and methods

### 3.1 Data

The initial sample for our study was selected from Chinese listed companies that reported on their CSR activities from 2010 to 2020. We chose public listed companies in China as our data source due to the reliability and consistency of their reported information, which is especially valuable given the challenges of data collection in emerging markets. The data on CSR activities of Chinese firms were extracted from the evaluation system of Hexun.com, a source increasingly utilized by researchers [103]. The Hexun.com evaluation system assesses firms’ performances in five distinct areas of CSR: responsibility towards shareholders, employees, suppliers, customers and consumers (collectively referred to as product responsibility), environmental stewardship, and social (community) responsibility. The score for community responsibility, in particular, is derived from the sum of public welfare donations and the proportion of income tax to total profits. For the purpose of our analysis and to derive broader insights, we focus specifically on charitable donations when defining community responsibility. Data on minimum wage increases were obtained from the Ministry of Human Resources and Social Security of the People’s Republic of China [104], while other essential firm-level characteristics were primarily sourced from the China Stock Market and Accounting Research (CSMAR) database. We use a panel data regression model, which is richer and more refined than the cross-sectional data from the questionnaire. However, this method also lacks timeliness, which motivates us to synthesize more methods for our study.

### 3.2 Definitions and measurements of main variables

Dependent variables: referring to the CSR measurement method of Kramer (2006, 2011) [16, 17] as well as the weight determination method, we creatively summed up the scores of environmental responsibility and community responsibility according to the weight to obtain the Response CSR score (RCSR). Similarly, the scores of product responsibility and customer responsibility were added up according to the weights to obtain the Strategic CSR score (SCSR). According to the calculation method of HeXun.com for calculating CSR scores, we multiplied strategic CSR and responsive CSR by 0.3 and 0.4, respectively, to distinguish the differences in their importance.

Independent variable: our independent variable was the maximum minimum wage standard of the province where the firms are registered in that year [104]. In fact, only Beijing and Shanghai have raised the minimum wage year by year after 2010. In the year when the minimum wage standard was not raised, although the labor cost might rise, it was not reflected by the minimum wage value of that year. In view of our focus on the minimum wage policy, in order to better abstract the general conclusion, we only considered the increase of human cost caused by the minimum wage policy.

Customer concentration-moderating variable: based on previous literature, we have constructed three different measures to measure the customer concentration level facing suppliers [37, 90]. All three indicators are based on the percentage of the firm’s major customers as a percentage of sales. Since December 2007, CSRC has asked firms to disclose the proportion of their top five customers in total sales. The data was derived from CSMAR and was expressed in empirical analysis by variables called “Customer concentration”.

CSR-sensitivity-moderating variable: the strategic value of CSR is likely lower in industries that are less CSR-sensitive—that is, industries where stakeholder support plays a marginal role for firms’ competitiveness and survival [38, 105]. A prime example of industries that are less CSR-sensitive are B2B industries. Because our research focused on the industry level in the sensitivity issue, this paper used the variable "CSR-sensitivity" in the empirical analysis.

### 3.3 Definitions and measurements of control variables

On the basis of consulting the previous literature related to CSR [23, 32, 96], this paper controls a series of firm characteristics that affect CSR investment.

In addition, the compensation of senior managers (Top managers salary) is also included in the indicators that may affect the effectiveness of human cost. The relevant data used the total compensation of the top three executives matched with the observation points in the CSMAR database.

Size was the natural logarithm of the book value of total assets.

ROA was the ratio of operating income before depreciation to the book value of total assets.

Tobin’s Q is the ratio of the market value of total assets to the book value of total assets.

The Cash holdings are the natural logarithm of the book value of total cash at beginning of year.

Leverage is the ratio of debt to the book value of total assets.

These covariates reflect the differences of firm size, profitability, investment opportunities, financing (leverage) and liquidity (Cash Holdings), which may affect the subsequent investment of strategic resources. The data are from CSMAR database.

To ensure that our results are not driven by outliers, we winsorized all control variables generated from the accounting data at the 1 percentile in each tail. Tables 1 and 2 summarize variable definitions, descriptive statistics and correlation analysis, respectively.

**Table 1:** Variable definitions.

Variable	Definitions	Data sources
**Top managers salary**	Executive compensation	CSMAR database
**Size**	Firm size = ln(assets)	
**ROA**	Return on assets	
**Tobin’s q**	The value creation ability of the firms	
**Cash holding**	The cash flow of the firm = (short term investments + cash)/total assets	
**Leverage**	Firms leverage	
**Philanthropy CSR**	Charitable donations	HeXun.com
**Customer CSR**	Corporate social responsibility for products	
**Environment CSR**	Environmental corporate social responsibility	
**Employee CSR**	Corporate social responsibility for employee	
**Minimum wage**	The minimum wage for the year in which the firm is registered	Ministry of Human Resources and Social Security of the People’s Republic of China
**Customer concentration**	The proportion of sales to the top five customers	CSMAR database
**CSR-sensitivity**	The codes belonging to CSR-sensitivity industries are 1, and the rest are 0	
**SCSR**	Strategic corporate social responsibility = 0.3* (Employee CSR + Customer CSR)*100	
**RCSR**	Responsive corporate social responsibility = 0.4* (Environment CSR + Philanthropy CSR)*100	
Minimum wage*CSR-sensitivity	The moderating effect of CSR sensitivity	
Minimum wage*Customer concentration	The moderating effect of customer concentration	

**Table 2:** Descriptive statistics and correlation analysis.

**Variables**	**1**	**2**	**3**	**4**	**5**	**6**	**7**	**8**	**9**	**10**
**1. SCSR**	1									
**2. RCSR**	0.787***	1								
**3. Minimum wage**	-0.180***	-0.164***	1							
**4. Customer concentration**	-0.072***	-0.125***	-0.0080	1						
**5. Top managers salary**	0.100***	0.124***	0.319***	-0.152***	1					
**6. Size**	0.222***	0.258***	0.239***	-0.170***	0.452***	1				
**7. ROA**	0.0050	0.0100	-0.0030	-0.022***	0.0060	-0.020***	1			
**8. Tobin’s q**	-0.014**	-0.025***	-0.021***	0.054***	-0.026***	-0.138***	0.034***	1		
**9. Cash holding**	0.0060	0.0050	-0.148***	0.039***	-0.044***	-0.183***	0.043***	0.049***	1	
**10. Leverage**	0.047***	0.049***	-0.017**	-0.0080	0.069***	0.248***	-0.089***	0.300***	-0.286***	1
**Mean**	138.900	264.000	1536.000	0.309	2102000.000	21.490	0.039	2.398	0.303	0.435
**SD**	228.900	281.000	372.600	0.223	1911000.000	1.371	0.823	14.200	0.205	0.315

* *p* < 0.10,

** *p* < 0.05,

*** *p* < 0.01

### 3.4 Empirical model and analyses

We then applied multiple regression analysis to examine the impact of minimum wages on strategic and tactical CSR. Next, we examined the moderating effect of adding customer concentration and CSR-sensitivity respectively. In order to alleviate the lag effect of minimum wage policy on CSR investment decisions, we used the lagged one-period model for regression analysis. The models are expressed as follows:

$${SCSR}_{it+1}=\alpha_{i}+\beta \times {Minimum\;wage}_{it}+\gamma^{,}X_{it+1}+\varepsilon_{it+1},$$ (1)

$${RCSR}_{it+1}=\alpha_{i}+\beta \times {Minimum\;wage}_{it}+\gamma^{,}X_{it+1}+\varepsilon_{it+1},$$ (2)

$${SCSR}_{it+1}=\alpha_{i}+\beta \times {Minimum\;wage}_{it}+\delta \times {Customer\;concentration}_{it}+\theta \times {Minimum\;wage}_{it}\times {Customer\;concentration}_{it}+\gamma^{,}X_{it+1}+\varepsilon_{it+1},$$ (3)

$${RCSR}_{it+1}=\alpha_{i}+\beta \times {Minimum\;wage}_{it}+\delta \times {Customer\;concentration}_{it}+\theta \times {Minimum\;wage}_{it}\times {Customer\;concentration}_{it}+\gamma^{,}X_{it+1}+\varepsilon_{it+1},$$ (4)

$${SCSR}_{it+1}=\alpha_{i}+\beta \times {Minimum\;wage}_{it}+\delta \times {CSR-sensitivity}_{it}+\theta \times {Minimum\;wage}_{it}\times {CSR-sensitivity}_{it}+\gamma^{,}X_{it+1}+\varepsilon_{it+1},$$ (5)

$${RCSR}_{it+1}=\alpha_{i}+\beta \times {Minimum\;wage}_{it}+\delta \times {CSR-sensitivity}_{it}+\theta \times {Minimum\;wage}_{it}\times {CSR-sensitivity}_{it}+\gamma^{,}X_{it+1}+\varepsilon_{it+1},$$ (6)

where i indexes firms; t indexes years; α_*i*_ are firm fixed effects; SCSR_*it*+1_ and RCSR_*it*+1_ stand for strategic and responsive CSR, respectively; Minimum wage_*it*_ is the minimum wage increase; X is the vector of control variables (Top managers salary, Size, ROA, Tobin’s q, Cash holding, Leverage) in the preceding year; Customer concentration_*it*_ and CSR–sensitivity_*it*_ represent customer concentration and CSR-sensitivity industries, respectively; ε is the error term. β is the coefficient indicating the effect of minimum wage increase, and θ represents the coefficient indicates the moderating effect.

## 4 Results

### 4.1 Main results

Our study included 16,450 samples. From Table 2, we also know that the correlation coefficient between key variables is less than 0.7, indicating that there is no multiple collinearity in the regression. The main results are presented in model 1 of Table 3 report estimates from the OLS regression specified in Eqs (1) and (2), respectively. As can be seen, different from our hypothesis 1, the minimum wage coefficients in model 1 and model 2 are both negative, which indicates that the investment in strategic and responsive CSR will be reduced. This was only consistent with adjustments for responsive CSR, but did not validate the role of maintenance for strategic social responsibility. However, from the absolute value of the coefficient (0.194<0.237, p < 0.01), we can see that the weakening effect of firms on strategic CSR is less than that on responsive CSR. In addition, we distinguish how different kinds of CSRs respond to the minimum wage in Table 4. The results show that customer responsibility is cut more in strategic CSRs and environmental responsibility is cut more in responsive CSRs.

**Table 3:** Main results and moderating effects.

Model	Model 1		Model 2		Model 3	
*Dependent variables*_*t+1*_	SCSR	RCSR	SCSR	RCSR	SCSR	RCSR
***Controls***_***t+1***_						
Top managers salary	-0.008***	-0.007**	-0.008***	-0.007**	-0.008***	-0.006**
	(-3.09)	(-2.28)	(-2.93)	(-2.15)	(-3.05)	(-2.25)
Size	29.367***	50.427***	28.998***	50.924***	28.494***	49.656***
	(6.92)	(9.96)	(6.47)	(9.31)	(6.74)	(9.76)
ROA	0.528	4.015	1.868	5.024	0.504	3.994
	(0.28)	(0.53)	(0.78)	(0.65)	(0.27)	(0.53)
Tobin’s q	0.618	0.614**	0.778	0.367	0.604	0.602*
	(1.60)	(1.99)	(1.58)	(1.05)	(1.57)	(1.96)
Cash holding	-52.381***	-58.471***	-52.696***	-49.270***	-51.272***	-57.491***
	(-3.84)	(-3.62)	(-3.62)	(-2.82)	(-3.77)	(-3.56)
Leverage	-1.977	-39.531***	6.684	-31.660***	-2.530	-40.020***
	(-0.33)	(-4.40)	(0.96)	(-3.54)	(-0.42)	(-4.45)
***Independent variable***						
**H1:** Minimum wage	-0.194***	-0.237***	-0.238***	-0.278***	-0.205***	-0.246***
	(-19.92)	(-20.70)	(-15.29)	(-15.60)	(-19.27)	(-19.66)
**H2:** Minimum wage* Customer concentration			0.119***	0.119***		
			(3.11)	(2.76)		
**H3:** Minimum wage*CSR-sensitivity					0.045**	0.040*
					(2.38)	(1.96)
Constant	-174.144**	-425.566***	-105.664	-375.503***	-156.407*	-409.901***
	(-2.02)	(-4.17)	(-1.12)	(-3.33)	(-1.83)	(-4.00)
*R* ^2^	0.081	0.077	0.088	0.080	0.082	0.077
F	67.835	73.406	50.806	54.840	60.033	64.291
*N*	16450	16450	14657	14657	16450	16450

*t* statistics in parentheses

* *p* < 0.10,

** *p* < 0.05,

*** *p* < 0.01

**Table 4:** Main results for different types of CSR.

Model	(1)	(2)	(3)	(4)
*Dependent variables*_*t+1*_	Strategic CSR		Responsive CSR	
	Customer CSR	Employee CSR	Philanthropy CSR	Environment CSR
***Controls***_***t+1***_				
Top managers salary	-0.0002***	-0.00005*	0.00002	-0.0002***
	(-3.83)	(-1.72)	(0.64)	(-3.29)
Size	0.545***	0.435***	0.669***	0.619***
	(6.25)	(7.06)	(8.83)	(6.42)
ROA	0.010	0.008	0.105	0.001
	(0.27)	(0.29)	(0.63)	(0.04)
Tobin’s q	0.010	0.010	0.005*	0.012
	(1.56)	(1.63)	(1.74)	(1.58)
Cash holding	-1.073***	-0.668***	-0.246	-1.216***
	(-3.66)	(-3.66)	(-1.03)	(-3.96)
Leverage	-0.180	0.113	-0.796***	-0.188
	(-1.48)	(1.11)	(-4.41)	(-1.40)
***Independent variable***				
Minimum wage	-0.0039***	-0.0025***	-0.0014***	-0.0045***
	(-19.40)	(-18.57)	(-10.42)	(-19.31)
Constant	-3.257*	-2.569**	-7.180***	-3.879**
	(1.767)	(1.249)	(1.530)	(1.934)
N	16450	16450	16450	16450
r2	0.079	0.071	0.016	0.087
F	68.072	55.645	22.116	66.692

*t* statistics in parentheses

* *p* < 0.10,

** *p* < 0.05,

*** *p* < 0.01

A look at the control variables provides additional insights into the determinants of CSR investment. In particular, larger and more profitable firms are more likely to maintain the two CSR investments. The same applies to firms that represent better profitability.

### 4.2 Moderating effect

In model 2 of Table 3, we examine whether customer concentration, which indicates the degree of customer richness, differentiation strategy and product diversification, alleviates or intensifies the process of adjusting CSR investment. Such firms build up reputation through past interactions and hence are less likely to benefit from CSR as a signal of trustworthiness.

**Fig 1:**
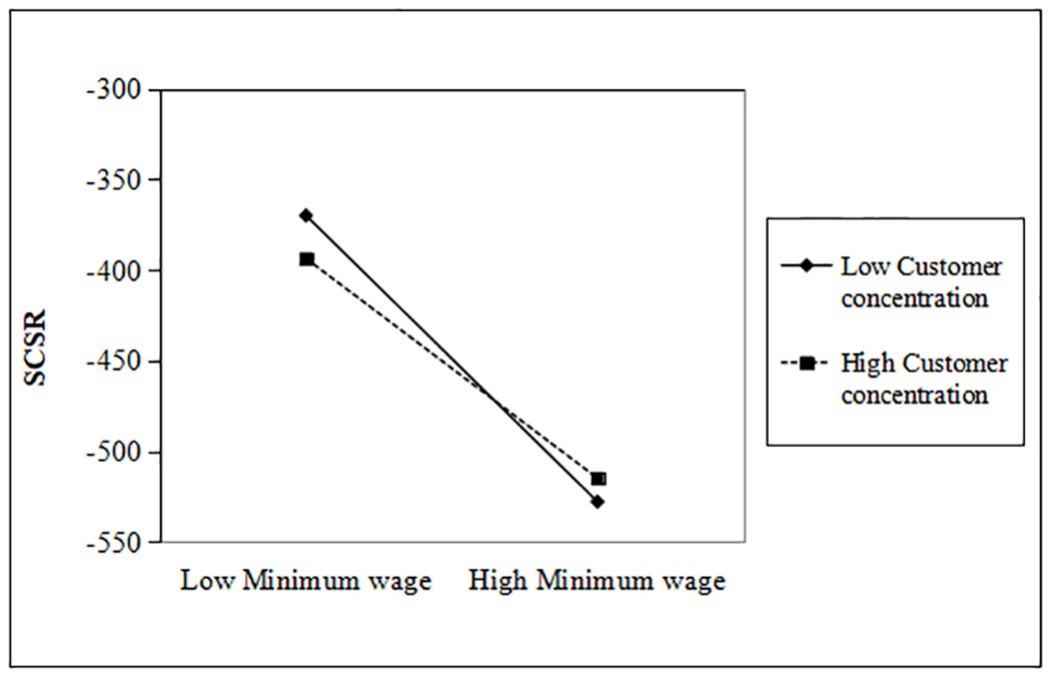
Moderating effect of customer concentration (SCSR).

**Fig 2:**
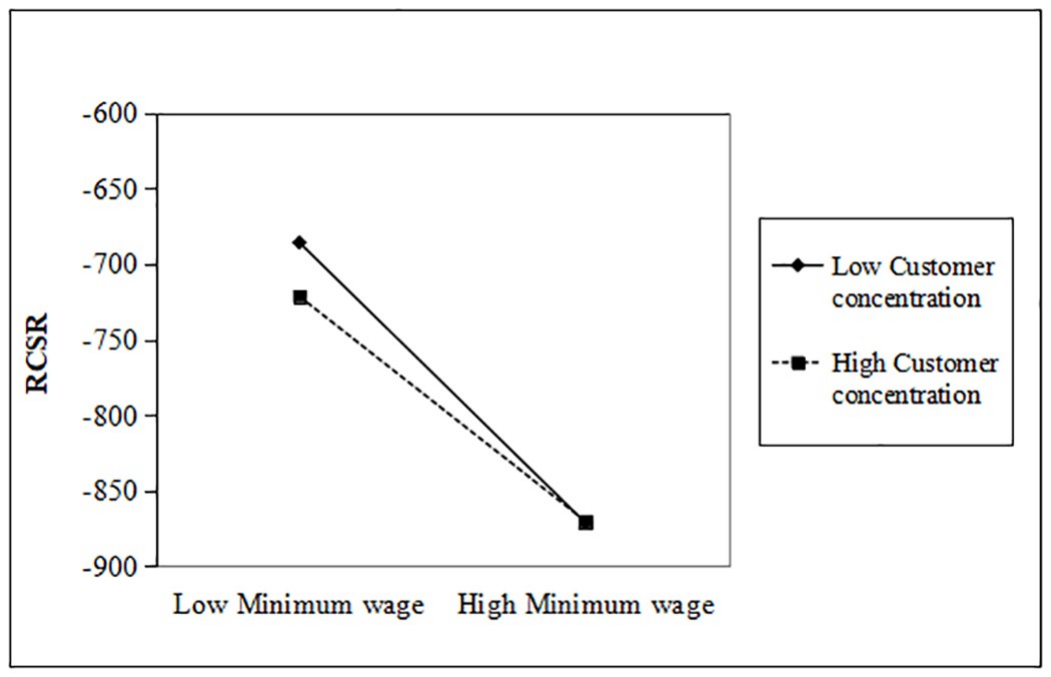
Moderating effect of customer concentration (RCSR).

To examine this hypothesis 2, we interact the Minimum Wage with Customer Concentration indicating whether “moderation effect”. As you can see, customer concentration significantly mitigates the decline in strategic and responsive CSR investments (θ = 0.119, p < 0.01). The moderating effect of customer concentration is more clearly expressed in Figs 1 and 2.

Next, we examine whether industries that are relatively sensitive to CSR may pay more attention to CSR investment, that is to say, when facing the pressure of rising costs, the reduction of CSR investment may be reduced. Hypothesis 3 mainly wants to explore the influence mechanism of CSR at the industry level. To test this hypothesis, we use the interaction term between Minimum wage and CSR-sensitivity to show whether CSR-sensitivity firms mitigate the decline in CSR investment.

**Fig 3:**
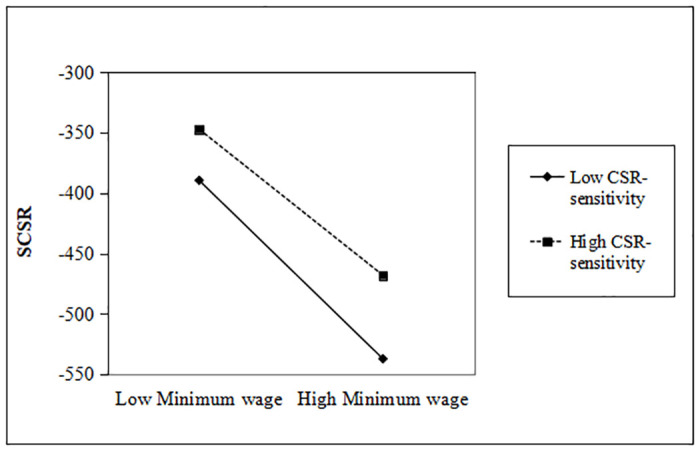
Moderating effect of CSR-sensitivity (SCSR).

**Fig 4:**
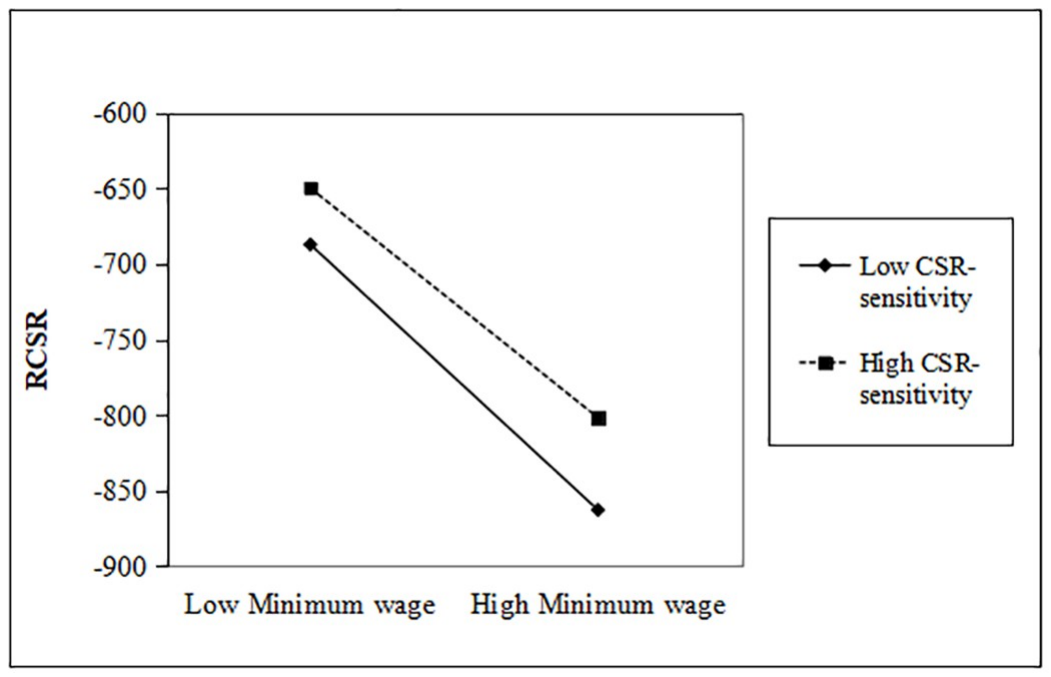
Moderating effect of CSR-sensitivity (RCSR).

As can be seen in model 3 of Table 3, we find that firms with high CSR-sensitivity have a significant weakening effect on the reduction of CSR. In particular, compared with reactive CSR, the downward trend of strategic CSR investment is significantly restrained (0.045>0.040, p < 0.05, p < 0.1). This finding is supportive of Hypothesis 3. Figs 3 and 4 respectively expressed the moderating effect of CSR-sensitivity.

### 4.3 Robustness tests

In order to verify the reliability of the above empirical conclusions, this paper conducts a robustness test. We changed dependent variables, independent variables and moderator variables by referring to previous practices [106, 107]. First of all, CSR is rated in Hexun.com, consisting of A, B, C, D and E, corresponding to CSR score of 80–100, 60–80, 40–60, 20–40 and below 20 respectively. We select the average score of each level, namely 90, 70, 50, 30 and 10, as the total CSR score of each firm, and then, according to the accounting method of HeXun.com, calculate the value of strategic and responsive CSR respectively according to the 30% of strategic CSR score (SCSR0) and 40% of responsive CSR score (RCSR0) to replace the dependent variable. Secondly, we use the sequential growth rate of minimum wage to replace the minimum wage value of each province to complete the substitution of independent variables [108]. Subsequently, we tested our model and found that our results did not change significantly, and the results still supported Hypothesis 1, as shown in Model 1 in Table 5. Third, we replace the continuous variable of customer concentration with dummy variable. The mean value of customer concentration above or equal to is denoted as 1, and the rest is 0. The adjustment of customer concentration is still significant after testing, as shown in Model 2 in Table 5.

**Table 5:** Robustness test—Main results and moderating effects.

Model	Model 1		Model 2		Model 3	
*Dependent variables*_*t+1*_	SCSR0	RCSR0	SCSR0	RCSR0	SCSR0	RCSR0
***Controls***_***t+1***_						
Top managers salary	0.0003***	0.0004***	0.0003***	0.0004***	0.0003***	0.0004***
	(6.34)	(6.34)	(6.28)	(6.28)	(5.80)	(5.80)
Size	0.142*	0.189*	0.143*	0.191*	0.023	0.031
	(1.72)	(1.72)	(1.74)	(1.74)	(0.27)	(0.27)
ROA	-0.229	-0.306	-0.233*	-0.310*	-0.223	-0.297
	(-1.64)	(-1.64)	(-1.65)	(-1.65)	(-1.64)	(-1.64)
Tobin’s q	-0.003	-0.003	-0.003	-0.003	-0.004	-0.005
	(-1.31)	(-1.31)	(-1.32)	(-1.32)	(-1.61)	(-1.61)
Cash holding	-0.267	-0.356	-0.264	-0.351	-0.153	-0.204
	(-0.95)	(-0.95)	(-0.94)	(-0.94)	(-0.55)	(-0.55)
Leverage	0.612**	0.816**	0.604**	0.805**	0.600**	0.800**
	(2.18)	(2.18)	(2.15)	(2.15)	(2.12)	(2.12)
***Independent variable***						
**H1:** Minimum wage growth rate	-4.333***	-5.778***	-4.882***	-6.510***	-4.240***	-5.653***
	(-11.86)	(-11.86)	(-9.66)	(-9.66)	(-11.71)	(-11.71)
**H2:** Minimum wage growth rate * Customer concentration dummy variable			1.232*	1.643*		
			(1.67)	(1.67)		
**H3:** Minimum wage growth rate * CSR-sensitivity					0.002***	0.002***
					(5.43)	(5.43)
Constant	16.314***	21.752***	16.404***	21.872***	18.249***	24.332***
	(9.20)	(9.20)	(9.26)	(9.26)	(10.19)	(10.19)
*R* ^2^	0.032	0.032	0.032	0.032	0.036	0.036
F	35.114	35.114	27.649	27.649	34.022	34.022
*N*	12833	12833	12833	12833	12833	12833

*t* statistics in parentheses

* *p* < 0.10,

** *p* < 0.05,

*** *p* < 0.01

Finally, in the study of industry heterogeneity, we find that the CSR of tourism industry is different from that of other B2C firms, that is, the CSR of tourism firms is more sensitive. Specifically, CSR in the tourism industry will have a great impact on the brand, and the reputation or image of the tourism industry is more important than that of other industries [109]. As tourism is a consumer discretionary industry, it is easy for consumers to move away from brands that ignore CSR and may switch to those with high CSR [110]. Therefore, in the B2C industry, the tourism industry will be more sensitive to CSR. Therefore, by referring to previous experience [106, 110, 111], we focused on exploring the industry heterogeneity of tourism companies in the B2C industry. We selected 41 listed Chinese tourism firms as the samples [111] of the New CSR-sensitivity of the tourism industry to replace the moderating variables of CSR-sensitivity. The New CSR-sensitivity value of firms belonging to the tourism industry is recorded as 1, while that of the other industries is recorded as 0. After testing, Hypothesis 3 was still supported, as shown in Model 3 in Table 5.

## 5 Future prospects

Our findings lay the groundwork for future research, particularly in developing a theoretical base rooted in empirical evidence. Leveraging our results, there is a potential to craft a comprehensive theory that explains how firms modulate their CSR investments in reaction to increases in labor costs. This theoretical model could be broadened to include responses to other types of cost increases, such as those related to implementing safety measures during the COVID-19 pandemic. However, such theoretical expansions would require additional empirical support. Specifically, a more detailed empirical examination of CSR’s four dimensions could reveal their theoretical underpinnings. For example, while our research indicates a reduction in responsive CSR, an area ripe for further exploration is the blurring lines between environmental responsibility and charitable donations. Future studies could investigate which CSR activities are being pared down and the rationale behind these choices. Undertaking this research challenge necessitates access to granular micro-level data on companies’ operations and strategic orientations, offering a rich vein of inquiry for future academic endeavors.

## 6 Conclusions

Our investigation into whether companies frequently adjust their CSR investments in the face of substantial minimum wage increases yields an affirmative conclusion. This aligns with existing literature that suggests a negative correlation between CSR investments and minimum wages [23, 32]. Utilizing the frameworks of cost stickiness and optimal distinctiveness, our study examines if and how managers might decrease CSR expenditure as a means to contend with rising labor costs. Our findings indicate that the approach to adjusting CSR initiatives—particularly between strategic and responsive aspects—differs greatly, influenced by varying institutional pressures [16, 17].

Additionally, our analysis points to high customer concentration serving as a mitigating factor against the reduction in managerial commitment to CSR investments, with a more pronounced decrease observed in strategic CSR activities. When considering the CSR sensitivity of an industry, our research proposes that companies operating within sectors highly attuned to CSR are likelier to minimize cuts to responsive CSR initiatives. However, insights derived from a three-period lag model reveal that industries with high CSR sensitivity exhibit reluctance towards slashing strategic CSR investments. This highlights the sustained significance of strategic CSR endeavors, underscoring their role in the comprehensive long-term strategy of firms.

## 7 Implications

Our research significantly expands the existing corpus on CSR by incorporating the dimension of minimum wage policies. While current studies on CSR largely focus on the roles of institutional pressures and corporate strategic orientations, the influence of minimum wage regulations on CSR decisions has been largely overlooked [68, 70, 71]. By applying the theory of cost stickiness, our study delves into the interplay between regional minimum wage standards and firms’ CSR activities, uncovering the mechanisms at play and their economic consequences [21, 57]. In contrast to previous studies [21, 57], we focus on analyzing the cost stickiness of strategic CSR and reactive CSR. We concluded that strategic CSR is more cost sticky. Therefore, specifically, the increase in minimum wage reduced CSR investment. For example, Guangdong Province, the center of China’s manufacturing industry, faces pressure to increase minimum wages year after year. In 2018, Guangdong Province increased the minimum wage to RMB 2,100 per month, which significantly affected many manufacturing companies. These companies have had to reduce their investment in CSR in response to rising wage costs. For example, some electronics manufacturing companies cut financial support for employee vocational training and health and welfare programs, while reducing investment in environmental protection projects [23]. Hence, our findings offer fresh perspectives on CSR through the lens of minimum wage legislation.

Furthermore, this study contributes to the ongoing discussions about the motivations driving CSR efforts among Chinese firms by merging systemic and strategic viewpoints. In previous studies [17], the discussion of CSR has been dominated by institutional and strategic theories. However, few studies have integrated these frameworks to examine the motivations behind CSR. In contrast to previous studies [17], we add to this gap, particularly by considering the impact of minimum wage laws. Utilizing Porter and Kramer’s CSR decision-making framework [17], our study distinguishes between responsive and strategic CSR, investigating the balance between institutional adaptation and strategic positioning through the theory of optimal distinctiveness. Our results meld institutional and strategic insights, enriching the understanding of CSR and extending the applicability of the theory of optimal distinctiveness.

Lastly, our research sheds new light on how minimum wage systems impact firm performance from a CSR perspective. The bulk of the literature on minimum wage policies concentrates on their macroeconomic outcomes, such as effects on poverty, employment, and income distribution, with limited exploration of their direct impact on firm performance [68, 70, 71, 112]. In contrast to previous studies [68, 70, 71, 112], by analyzing how minimum wage regulations influence CSR and, in turn, affect a firm’s ability to secure government subsidies and compete in the product market, our study reveals a novel mechanism through which minimum wage policies can affect firm performance, offering a unique contribution to the understanding of the interconnections between labor economics and corporate strategy.

## 8 Recommendations

Our study’s practical implications are manifold. First, it offers a nuanced evaluation of the impacts stemming from the implementation of China’s minimum wage system, viewed through the CSR prism. Since the nationwide adoption of the Firm Minimum Wage Regulations in 2004, there has been a marked improvement in labor protection and income distribution in China [66, 70, 113]. Although the minimum wage system plays a critical role in safeguarding workers’ rights, it concurrently escalates labor costs for businesses. The concern for policymakers extends beyond the macroeconomic repercussions of rising minimum wages to include the nuanced understanding of how such policies influence behaviors and performance at the firm level. Our research sheds light on the ways minimum wage adjustments affect CSR and its economic ramifications, offering policymakers a scientific foundation to gauge the firm-level impacts of minimum wage policies. This insight is crucial for refining and enhancing the minimum wage system reforms, providing both theoretical insight and policy direction. Similar to the minimum wage, the COVID-19 epidemic and the payment of occupational insurance will also increase the cost of businesses. In addition, industry competition and consumer preferences also affect firms’ CSR investments. These additional factors can further enrich our future research in the future.

Secondly, our findings furnish theoretical and practical advice for Chinese companies amidst economic transition on how to refine CSR efforts, taking into account both systemic and strategic factors [23, 66, 67]. The challenge of aligning institutional responses with strategic objectives in CSR initiatives is significant in management practice. In the shifting economic environment of China, where the government is a key source of legitimacy and resources, it is imperative for managers to strategically navigate social responsibility. This involves crafting CSR practices that not only reflect the firm’s distinct attributes but also cater to institutional norms and expectations. It necessitates the development of a strategic CSR framework that is custom-fit to the company’s needs while also bolstering the firm-government relationship to enhance legitimacy and access resources. Our findings underscore the importance of achieving a dynamic equilibrium between responsive and strategic CSR, providing valuable empirical insights and guidance for Chinese businesses striving to meet both institutional and strategic demands during this period of transition.

## 9 Limitations

**Fig 5:**
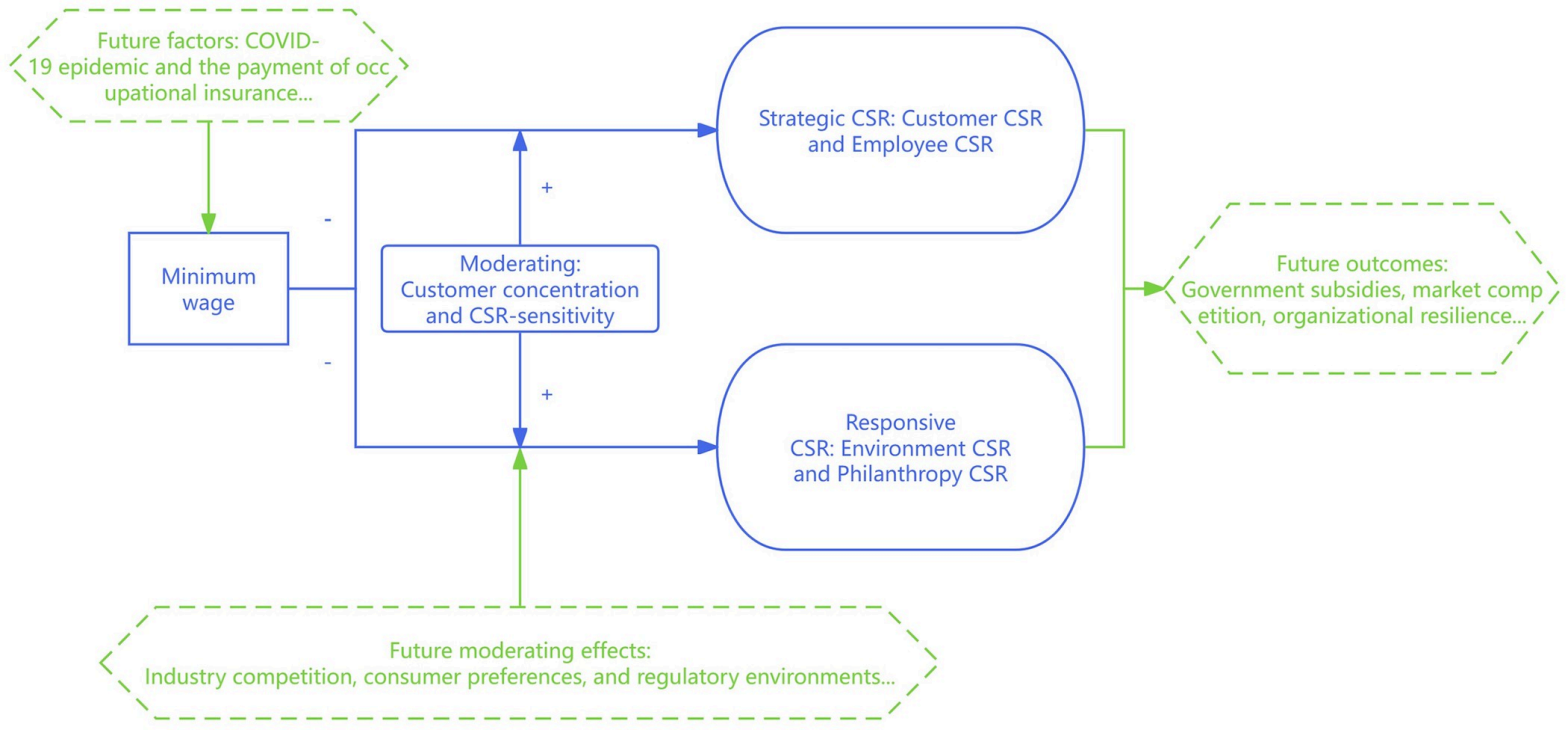
This study and possible future studies.

Our study conducts an in-depth analysis of how firms internally adjust their investments, recognizing that while the adaptation to external changes is a well-trodden area in existing literature, our research may not completely capture these external dynamics. Furthermore, the mandatory reporting of CSR activities by Chinese listed companies only started in 2010, resulting in a lack of data from earlier periods. As a result, our analysis does not fully examine the impact of minimum wage policies on the evolution of CSR practices over time. Utilizing regression analysis within the framework of current theories, this study endeavors to elucidate the intricate effects of minimum wage adjustments on firm-level decision-making. In doing so, it aims to foster further research and contribute to the development of related theories. In addition, we recognize that our data do not adequately address the impact of the COVID-19 epidemic. Besides, our variable “CSR-sensitivity”, which is relatively simple to measure, can be measured using more complex continuous variables in the future. This could serve as additional research in the future. The structure of this study and possible future studies is shown in Fig 5.

## Supporting information

S1 File Dataset. This is the dataset used in the analysis.
(CSV)
